# The pervasive role of biological cohesion in bedform development

**DOI:** 10.1038/ncomms7257

**Published:** 2015-02-06

**Authors:** Jonathan Malarkey, Jaco H. Baas, Julie A. Hope, Rebecca J. Aspden, Daniel R. Parsons, Jeff Peakall, David M. Paterson, Robert J. Schindler, Leiping Ye, Ian D. Lichtman, Sarah J. Bass, Alan G. Davies, Andrew J. Manning, Peter D. Thorne

**Affiliations:** 1 School of Ocean Sciences, Bangor University, Menai Bridge, Anglesey LL59 5AB, UK; 2 Sediment Ecology Research Group, School of Biology, University of St Andrews, Fife KY16 8LB, UK; 3 Department of Geography, Environment and Earth Sciences, University of Hull, Hull HU6 7RX, UK; 4 School of Earth and Environment, University of Leeds, Leeds LS2 9JT, UK; 5 School of Marine Science and Engineering, Plymouth University, Drake Circus, Plymouth PL4 8AA, UK; 6 National Oceanography Centre, Joseph Proudman Building, 6 Brownlow Street, Liverpool L3 5DA, UK; 7 HR Wallingford, Wallingford OX10 8BA, UK

## Abstract

Sediment fluxes in aquatic environments are crucially dependent on bedform dynamics. However, sediment-flux predictions rely almost completely on clean-sand studies, despite most environments being composed of mixtures of non-cohesive sands, physically cohesive muds and biologically cohesive extracellular polymeric substances (EPS) generated by microorganisms. EPS associated with surficial biofilms are known to stabilize sediment and increase erosion thresholds. Here we present experimental data showing that the pervasive distribution of low levels of EPS throughout the sediment, rather than the high surficial levels of EPS in biofilms, is the key control on bedform dynamics. The development time for bedforms increases by up to two orders of magnitude for extremely small quantities of pervasively distributed EPS. This effect is far stronger than for physical cohesion, because EPS inhibit sand grains from moving independently. The results highlight that present bedform predictors are overly simplistic, and the associated sediment transport processes require re-assessment for the influence of EPS.

## Abstract (web summary)

The role of cohesion is vital to our understanding of how sedimentary bedforms evolve. Here, the authors show that microorganisms within the sediment affect cohesion and demonstrate that ripples can take up to one hundred times as long to develop when extracellular polymeric substances are present.

## Graphical abstract

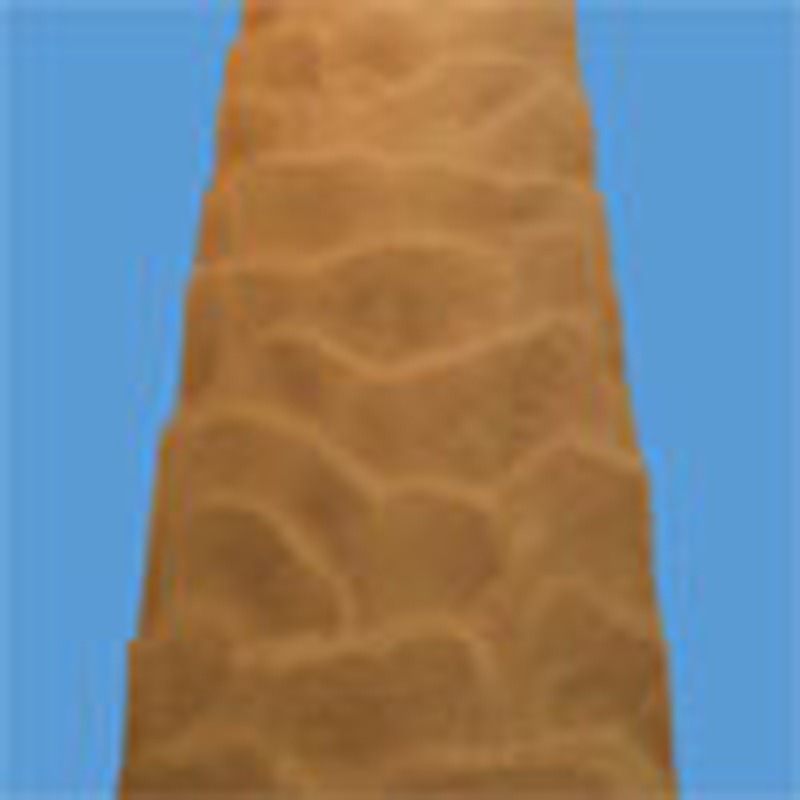

In aquatic environments, most natural sediment is composed of non-cohesive sands, physically cohesive muds and biologically cohesive extracellular polymeric substances (EPS)^1^, generated by microorganisms that are ubiquitously present^2^. Yet, most practical predictions of sediment transport are based almost exclusively on non-cohesive sand^3^. Active zones of sediment transport in aquatic environments exhibit a range of dynamic sedimentary bedforms, which are key controls on scour, erosion and deposition. Most research concerning bedform prediction has also focused on non-cohesive sands^4^,^5^, and whilst a substantial body of literature on the effect of physical and biological cohesion on erosion exists^6^,^7^,^8^, this is mainly for mud-dominated systems rich in EPS, where bedforms are unlikely to form^9^. Thus, there is very little research on the formation and dynamics of bedforms in biologically cohesive sediment^10^. However, for physical cohesion, recent steady-flow experiments over beds of fine sand and kaolin clay^11^ have demonstrated that mud content reduces bedform height and that for up to 12% mud the presence of bedforms provides efficient winnowing of the mud from the bed.

Biological cohesion occurs where organic molecules (extracellular polymeric substances, EPS) are secreted by organisms that inhabit natural sediments. Important sources of EPS are sediment bacteria and microphytobenthos (mainly diatoms) that form biofilms at the sediment surface. The EPS matrix produced by these microbial assemblages prevents sand grains from moving independently by forming bonds between them^12^. Laboratory experiments and theoretical analysis, involving currents over sand and naturally occurring EPS, have recently demonstrated that surficial biofilm development completely suppresses sediment transport until flow velocities are sufficiently high to cause catastrophic failures in the biofilm^13^,^14^.

Surficial biofilms can have concentrations of EPS up to 5% (dry weight) in intertidal muds^15^ and ~1% in freshwater muds^16^. Lower concentrations of EPS (0.01–0.1%) have been found pervasively distributed in sandy muds and sands with low mud content^10^,^17^,^18^, as shown in the vertical profiles of EPS (for example, Fig. 1a) from the Dee Estuary, UK. Figure 1b shows a scanning electron microscope image from the bed surface of a sandy site in the Eden Estuary, UK. Despite low levels of EPS (0.027–0.08%), the polymer bonds between sand grains are still clearly visible in the form of bridging structures. Such structures highlight that although the sand grains may not be prevented from moving independently, they will be inhibited from doing so where pervasive background EPS are present^2^,^12^.

The present work describes laboratory experiments conducted in a 10-m long, 0.3-m wide, recirculating laboratory flume^11^ where a 35-mm-thick sediment bed, composed of mixtures of fine well-sorted sand (median grain size, *D*_50_, of 0.148 mm) and different quantities (Table 1) of EPS (from 0 to 1% by weight), was created to examine the influence of pervasive biological cohesion on bedform development. Xanthan gum, which is a bacterial polymer used in the food industry^19^, was used as a proxy in the laboratory for naturally occurring EPS^20^. Flow velocity was set to be above the critical shear-stress threshold for sediment movement and was measured using Ultrasonic Doppler Velocimetry Probes (UDVPs)^11^. Bed morphology was quantified from time-lapse photography, permitting calculation of bedform dimensions and migration rates. The duration of each test varied depending on how quickly the bedforms reached equilibrium or whether they developed at all. The flow was initiated and ripples allowed to develop with the cameras continuously recording bedform evolution. UDVP measurements were taken periodically as the bedform and flow characteristics varied. Pre- and post-test bed samples were taken to determine the fraction of EPS remaining in the ripples after the test (Table 1). The EPS measurements are based on the carbohydrate content^21^, using the standard Dubois assay^22^. This work thus provides the first systematic set of experiments to study the effect of the pervasive ‘background’ EPS on the development of non-cohesive bedforms, as opposed to the much larger and localized accumulations of EPS associated with biofilms^14^.

## Results

### Abiotic case

Similar to previous work in clean sands, the results show that in the abiotic case (Fig. 2), once initiated, the bedforms grew in length and height, and reached equilibrium in less than 2 h. The bedform dimensions tended to increase in scatter as equilibrium was reached as bedforms changed in character from two-dimensional to three-dimensional^4^,^23^ (Fig. 3a). The equilibrium height, *H*_e_, and wavelength, *L*_e_, were 12±4 mm and 106±24 mm, respectively, classifying them as current ripples^4^. The development times (time to reach 90% of equilibrium) for height, *T*_H_, and wavelength, *T*_L_, were 1.1 and 1.3 h, respectively. These results are in good agreement with other clean sand results^11^, *H*_e_=16 mm, *L*_e_=116 mm, *T*_H_=1.06 h and *T*_L_=1.35 h, which involved sand with a similar *D*_50_. These values are also in good agreement with predictive formulae^5^, *H*_e_=14 mm, *L*_e_=112 mm, *T*_H_=0.65 h and *T*_L_=1.08 h, reinforcing the concept that the median grain diameter is the main controlling factor in the formation of these bedforms^4^.

### Effect of EPS on bedform dimensions

For the 1% EPS case, the bed remained featureless (Fig. 3c). The higher critical shear stress required to move sediment was evidence of the bed stabilizing effect of high, biofilm-equivalent, levels of EPS content^6^,^14^. Figure 4 shows the effect of increasing the EPS content in the bed from 0 to 0.125%, with 0.125% determined as the upper limit for bedform formation (Table 1). As with the abiotic (0% EPS) case, scatter tended to increase as equilibrium was approached. The main effect of the EPS was to dramatically increase the development time and also the time at which the bedforms first appeared (*t*_i_=0.1–7.9 h), even for the comparatively small amounts of EPS used (0–0.125 %). Thus, a two orders of magnitude increase in bedform initiation time was observed across this EPS range. This is a much stronger effect than for physical cohesion, where *t*_i_ does not increase significantly below a mud content of 16% (ref. 11).

The 0.125% test had not reached equilibrium after 9 h, and the bedforms were still two-dimensional (Fig. 3b)^4^. However, most of the other runs appear to be close to equilibrium, as their final dimensions fell within the scatter of the abiotic case and the ripples were three-dimensional. In fact, there was only a modest improvement in the least-square fits between assuming that *H*_e_ and *L*_e_ were the same as the abiotic case and finding *T*_H_ and *T*_L_ (abiotic dimensions fit (ADF), see Methods), versus determining all four parameters non-linearly (non-linear fit (NLF)). The former, which is depicted in Fig. 4, results in *T*_H_=1.1–115.2 h and *T*_L_=1.3–92.2 h for EPS contents of 0–0.063% (Table 1). Thus, the development time also increased by two orders of magnitude as EPS content increased from 0 to 0.063%. This is again a far stronger effect than for physical cohesion, where *T*_H_ and *T*_L_ show no significant change up to mud contents of 12% (ref. 11).

### Effect of EPS on transport rates

The transport rates and cumulative transport associated with ripple migration were calculated for each test where the ripple dimensions were calculated (Fig. 5; see methods section for details). The migration rates on which the sediment transport rates are based are consistent with previous migration rate data^24^. Figure 5 shows that initial EPS content has a large effect on the sediment transport. The transport rate decreases with increasing EPS content (Fig. 5a). This reduction is particularly apparent for the cumulative transport (Fig. 5c). Figure 5b reveals that all the transport rates collapse onto one curve when initiation time is subtracted and the time is normalized by *T*_H_. This is a reflection of the fact that the migration rate is primarily a function of ripple height and the transport rate is the product of the migration rate and the ripple height.

## Discussion

**Figure 1:**
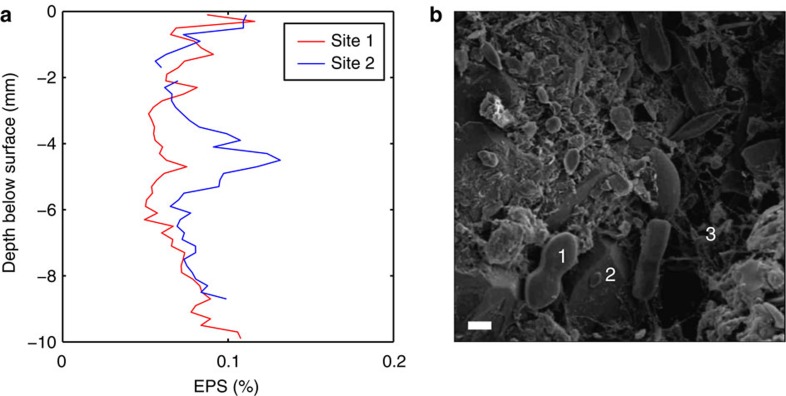
Extra polymeric substances in the field. (**a**) Vertical profiles of extra polymeric substances (EPS) taken from the Dee Estuary, UK^36^, in the spring where the median grain diameter, *D*_50_, was 0.233 mm. (**b**) Scanning electron microscope image of a sediment surface sample taken from the Eden Estuary, UK^15^, in the autumn, *D*_50_=0.277 mm and the EPS range was 0.027–0.08%. The scale bar is 10 μm. Here, the smaller particles are diatoms (1), the larger particles are sand grains (2) and the bridging structures are strands of EPS (3). See Supplementary Figs 1 and 2, Supplementary Table 1 and Supplementary Notes 1 and 2 for information about the field sites.

**Figure 2:**
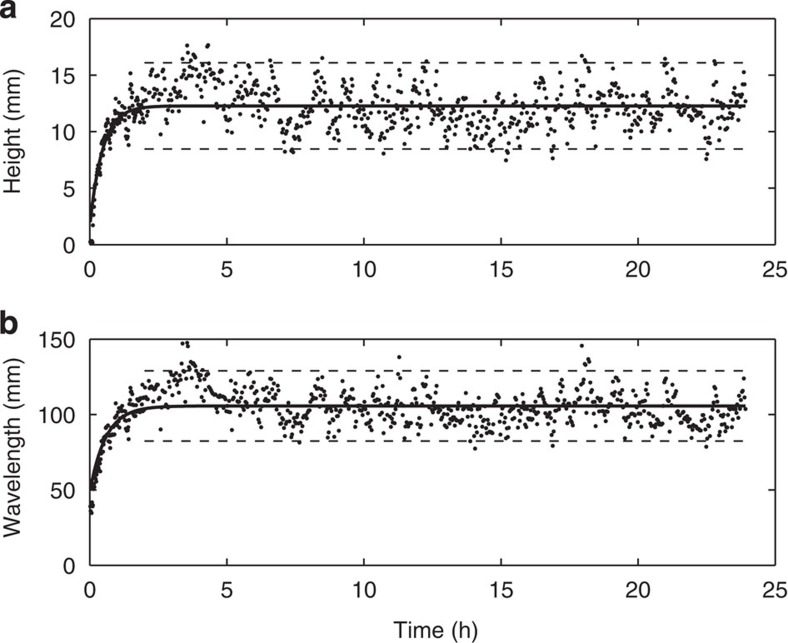
Ripple dimension development curves for the abiotic case. Ripple height (**a**) and wavelength (**b**) plotted against time. Solid line shows the fit to equation (1) (*T*_H_=1.1 h and *T*_L_=1.3 h); equilibrium dimensions (*H*_e_=12 mm and *L*_e_=106 mm) and 95% confidence intervals (dashed lines) are based on all values after 2 h.

The fraction of the initial EPS remaining after the end of each experiment (Table 1) demonstrates that as EPS concentration declines there is the tendency for all bedforms to evolve towards the equilibrium height and wavelength of abiotic bedforms. This indicates that the temporal increase in height and wavelength of the bedforms is caused by the gradual winnowing of the EPS from the sand. Most of the EPS is winnowed in 7–9 h. While it might seem that this is related to the ‘turnover’ associated with bedform migration^25^, EPS is still winnowed when the bedforms are poorly developed (0.125% case). This implies that the rate of removal of EPS from the bed is limited by the efficiency of the winnowing process on a flat surface rather than on a rippled surface, as indicated by the increase in initiation time with EPS content. Once ripples begin to develop the limiting factor becomes the supply of winnowed sediment from the troughs, hence the increase in development time with EPS content. This winnowing timescale of 7–9 h for EPS is an order of magnitude longer than for mud in identical experiments with sand–mud mixtures (for mud contents up to 18%)^11^. This finding matches comparisons between sand–mud and our sand–EPS experiments, where the time of first appearance and the development time of the bedforms are much longer in the sand–EPS case. These differences, which also affect the corresponding transport rates, reflect the fact that EPS binds directly to the sand grains (Fig. 1b), rather than acting as discrete particles or particle clusters as muds do in sand–mud mixtures^26^. The timescale for achieving equilibrium forms was of the order of half a tidal cycle (~6 h) for the 0.016% EPS case, and much greater for 0.031% and higher EPS levels, therefore indicating that in mixed intertidal sediments bedforms will be far smaller in terms of both height and wavelength than those in abiotic sand–mud mixtures.

Given the evidence for winnowing of EPS in our experiments, and that of mud in previous experiments^11^, the presence of mixed sediment in many coastal and estuarine environments appears paradoxical. Clearly, there are processes that act to counter the progressive winnowing by unidirectional flows. In part, the slow timescale of winnowing relative to the tidal cycle will aid long-term maintenance of EPS within the system, as will the continual production of new EPS by microbial assemblages inhabiting the sediment. EPS can be produced at up to 20 times the microbial biomass per hour^27^, where biomass is quantified by the Chlorophyll a (Chl a) concentration. Thus, for a Chl a concentration of 0.001% (ref. 10), this results in 0.02% of EPS per hour, or 0.1% of EPS over 5 h. This may be a conservative estimate because it neglects microbial mineralization and diatom locomotion^27^,^28^. In addition, EPS and mud are present in the water column and often form flocules^2^, which settle out more readily when sand is also in suspension^29^. In natural settings, EPS and mud can be re-incorporated into the bed by bioturbation^30^ (bedform degradation can occur in 4–6 h under sub-threshold conditions)^31^ and through subsurface ‘pumping’^25^ (this effect is weaker, as replenishment would take a day under sub-threshold conditions, see Methods). All of these factors that counter-act winnowing will cause bedform development to be slowed down even further, such that the effect of EPS will tend to be strengthened in the field.

As EPS is ubiquitous in the natural environment^2^,^12^ and our experiments demonstrate that only very small amounts are required to produce an effect (<0.125%), there are likely to be far-reaching consequences for bedform development. Indeed, the natural variability in bedform properties and the often unsatisfactory performance of bedform predictors for assumed abiotic sand are likely to be related to small amounts of EPS; understanding of these effects requires an interdisciplinary approach between sedimentologists and microbiologists.

These laboratory experiments on the effect of biological cohesion on bedform dynamics have shown that very small amounts of pervasively distributed EPS are sufficient to produce a substantial change in small-scale bedform development. The development time and time of first appearance both increase by two orders of magnitude for EPS contents increasing from 0 to 0.063% and 0 to 0.125%, respectively. This effect is far stronger than for the physical cohesion associated with muds mixed into sands^11^, because of differences in the binding of grains between sand–mud and sand–EPS mixtures. In the case of biological cohesion, bedform formation is drastically slowed down, because EPS inhibit the grains from moving independently.

This work demonstrates the importance of biological cohesion compared with physical cohesion in bedform formation. The presence or absence of bedforms in biologically active sediments is important in determining the bed roughness^1^ on larger spatial scales and for morphological calculations in modern and ancient environments. Thus, the large increase in both the development time and the time of first appearance of the bedforms relative to abiotic sand, as well as the large decrease in sediment transport, are crucial for sediment transport modelling in the natural environment. A greater understanding of the biological processes that influence sediment transport is required in order to better parameterize these effects in future models of aquatic environments.

## Methods

### Preparation of the bed

The mixes are listed in Table 1 and range from 0 to 1% EPS (xanthan). The sand and xanthan (which is in a dry powder form) were mixed in one of two ways: (i) weighing the sand and xanthan dry, mixing these and then adding water, one part freshwater to four parts sand/xanthan (listed as ‘D’) or (ii) wet by diluting the 1% mix by doubling the amount of sand for each subsequent test (listed as ‘W’). Method (ii), which is approximate, was used to determine the EPS content that was of most interest for ripple formation and method (i), which is exact, was used to determine ripple dimensions. In both cases, the method resulted in a slurry, which was spread evenly in the flume to make a flat bed with a thickness of about 35 mm and length of about 8 m. The flume was then filled with the required amount of freshwater and left for 24 h before the test was conducted.

### Instrumentation

**Figure 3:**
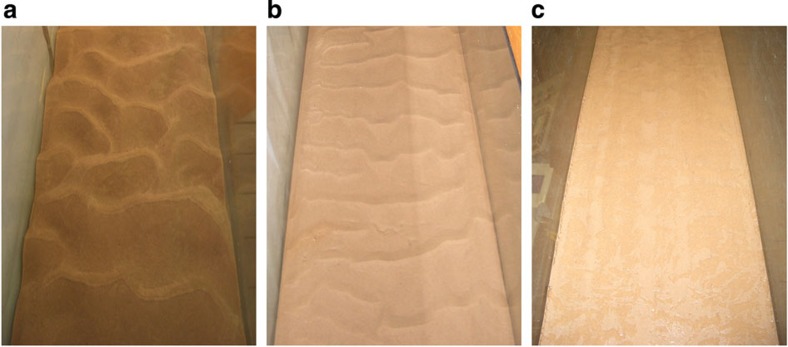
Photographs of post-test ripples. (**a**) Test 1 (0% EPS), (**b**) test 6 (0.125%) and (**c**) test 2 (1%), looking in the flow direction along the flume. The width of the flume is 300 mm.

**Figure 4:**
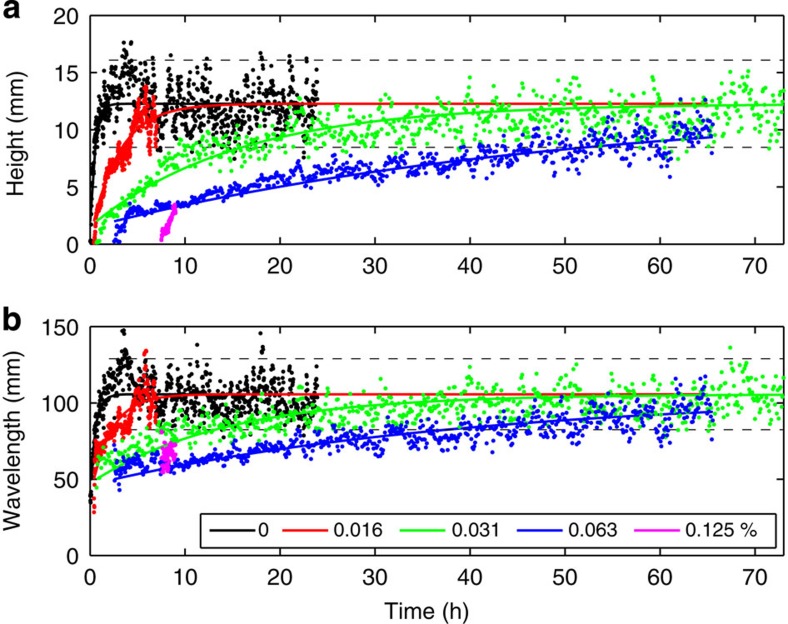
Ripple dimension development curves for the biotic cases. Ripple height (**a**) and wavelength (**b**) plotted against time for various initial EPS contents. Lines are based on fitting to equation (1) (*t*_i_=0.1, 0.4, 1, 2.7, 7.9 h for initial EPS=0, 0.016, 0.031, 0.063, 0.125%, *T*_H_=1.1, 6.9, 35.5, 115.2 h and *T*_L_=1.3, 5, 33, 92.2 h for initial EPS=0, 0.016, 0.031, 0.063%) assuming abiotic equilibrium dimensions, *H*_e_=12 mm, *L*_e_=106 mm.

The flow velocity was measured using 2 MHz UDVPs at 10 different heights above the bed (looking horizontally into the flow along the centre-line). The UDVPs had a measurement window of about 100 mm (128 bins) and a duration of about 90 s (250 time instants). The procedure for analysing the UDVP data is as described previously^11^. Ripple development on the glass side wall of the tank was characterized using two time-lapse cameras focusing on adjacent patches of the bed. It was assumed that the side wall image was representative of the rippled bed as a whole and examination of the bed after the test (Fig. 3a–c) confirmed this (that is, there was no edge effect visible in the ripple pattern).

### Analysis of the time-lapse images

The time-lapse images were analysed using a MATLAB script developed for the purpose. The steps involved in the process to determine the height and wavelength of the ripples, which is similar to that described by van der Mark *et al*.^32^, are outlined below:

- Pick out the rippled surface on the glass by specifying an appropriate contour interval in the green colour band of the image.
- Smooth the profile using a running mean.
- Select the maxima and minima based on the first derivative being zero and the sign of the second derivative.
- Determine wavelength and height based on the length of the line between two consecutive minima and the perpendicular distance from this line to the maximum between these minima, respectively.
- Take an average of all ripples for a given time from both cameras.

### Time development of ripples

The rate of growth of the ripple dimensions is proportional to their difference from some final equilibrium value. So it can be anticipated^4^ that the time development of ripple dimensions can be expressed generically as


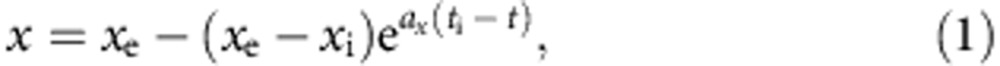


where *x* is the variation in the dimension (height, *H*, or wavelength, *L*) with time, *t*, *x*_e_ is the equilibrium dimension, *x*_i_ and *t*_i_ are the initial dimension and time (*x*(*t*_i_)=*x*_i_), *α*_*x*_=log(10)/*T*_*x*_ and *T*_*x*_ is the time taken for the dimension to reach 90% of its equilibrium value. Here *t*_i_ corresponds to the time of first significant appearance of bedforms on the sidewall.

### Natural variability of abiotic ripples

For the abiotic ripples, the 95% confidence intervals correspond to standard deviations of about 16% and 9% relative to *H*_e_ and *L*_e_, respectively. This is smaller than the standard deviations found previously^32^, which were typically about 50% of the equilibrium, based on very long record lengths. However, if individual ripples are averaged together for all times after 2 h, rather than the mean at each time, the equilibrium values are similar, but the standard deviations rise to 46% and 33%, respectively. Although this assumes that all ripples from different times may be grouped together and are independent, it none-the-less demonstrates that the present measurements are capturing the natural variability in ripple dimensions seen by other researchers^32^. It also further justifies the present experimental setup, including determining the ripple dimensions from the side wall and the width of the flume used.

### Dimensions and development times for biotic ripples

When all four parameters are determined, the NLF results in *H*_e_=13, 11 and 11 mm, *T*_H_=8.2, 27.1, 92.2 h (*r*^2^=0.88, 0.76, 0.88) and *L*_e_=110, 101 and 100 mm, *T*_L_=5.9, 24.3, 76.9 h (*r*^2^=0.72, 0.60, 0.75) for 0.016%, 0.031%, 0.063% EPS, respectively. When the equilibrium dimensions are assumed to be the same as the abiotic case, the ADF results in *T*_H_=6.9, 35.5, 115.2 h (*r*^2^=0.88, 0.74, 0.88) and *T*_L_=5.0, 33, 92.2 h (*r*^2^=0.71, 0.61, 0.76) for 0.016%, 0.031%, 0.063% EPS, respectively. The NLF dimensions are within the scatter of the abiotic case (*H*_e_=12±4 mm, *L*_e_=106±24 mm). Also, there are only small differences in the goodness-of-fits between the NLF and ADF, and in some cases the latter improves the fit.

### Sediment transport associated with ripple migration

The sediment transport per unit width associated with ripple migration was calculated using the following steps:

- For each camera, the ripple migration rate, *c*_m_, was determined by cross-correlation between two consecutive images^33^.
- The transport rate for each pair of images was determined using 0.5*ρ*_s_ *c*_0_ *c*_m_ *H*, where *ρ*_s_ is the density of sand (=2.65 g cm^−3^) and *c*_0_ is the solid bed volumetric concentration (=0.6).
- The transport rates for each camera were averaged together to give a value for each time instant.
- The cumulative transport was calculated by integrating the transport rates up to each time instant and multiplying by the width of the tank (300 mm).

### Time for complete trapping of a colloid in the bed below the threshold of motion

**Figure 5:**
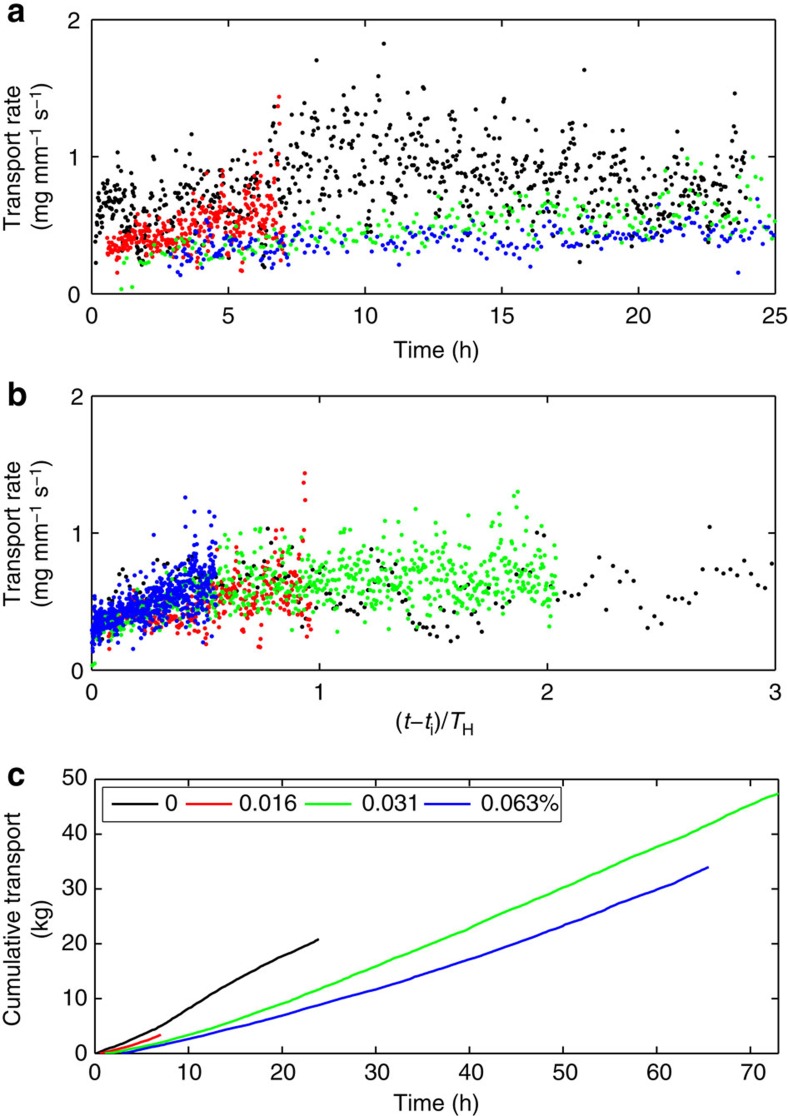
Time development of sediment transport associated with ripple migration. Sediment transport per unit width of the flume plotted against time (**a**) and non-dimensional time (**b**), and, cumulative transport plotted against time (**c**), for the various initial EPS contents. The legend for all plots is shown in part **c**.

Although turnover dominates when ripples migrate, the pumping mechanism associated with Darcy’s Law can still operate when the flow is below threshold as can occur in a tidal environment. The depth-averaged velocity corresponding to the threshold of motion^3^ is *U*=285 mm s^−1^, in the absence of ripples. For complete trapping of a colloid in the bed as given by Fig. 5a^34^ and a depth-averaged current of 250 mm s^−1^, which is the closest tabulated value available, the time taken for the concentration in the bed to reach 90% of its value in the flow, *t*_90_, requires that *ku*_m_*t*_90_/(1–*c*_0_)=36. Here *k*=2*π*/*L* and *u*_m_ is the maximum induced pore water velocity^34^, which is given by *u*_m_=*kKh*_m_, where *K* is the hydraulic conductivity^35^ and *h*_m_ is the half amplitude dynamic head, given, respectively, by


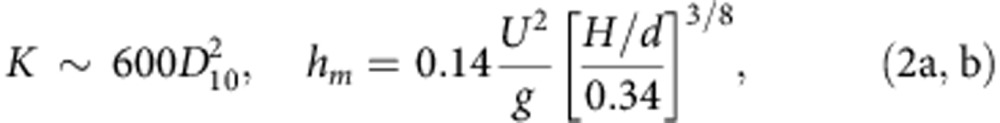


where *K* is in mm min^−1^, *D*_10_ is in mm, *g* is the acceleration due to gravity and *d* is the water depth. In the present experiments, *D*_10_=0.101 mm, so *K* is given by 6 mm min^−1^. For test 11, *H* and *L* are taken as their equilibrium values (12 and 106 mm), *d*=252 mm and *U* is taken as 250 mm s^−1^. This gives *u*_*m*_=0.15 mm min^−1^ and therefore *t*_90_=26.7 h (~1 day).

## Author contributions

J.H.B., D.R.P., S.J.B., D.M.P., P.D.T., A.G.D., A.J.M., J.P. and R.J.S. conceived the study; J.M. and J.H.B. undertook the experiments; J.H. and R.J.A. undertook the carbohydrate analysis; J.H. processed the SEM image; J.M. undertook the ripple and UVP analysis; J.M. wrote the paper with significant contribution from all authors.

## Additional information

**How to cite this article**: Malarkey, J. *et al*. The pervasive role of biological cohesion in bedform development. *Nat. Commun.* 6:6257 doi: 10.1038/ncomms7257 (2015).

## Supplementary Material

Supplementary Information Supplementary Figures 1-2, Supplementary Table 1 and Supplementary Notes 1-2.

## Figures and Tables

**Table 1:** Parameters for the sand–EPS experiments.

**Test**	**EPS (%)**	**Water depth (mm)**	***U***_**i**_**(mm** **s**^−**1**^**)**	***U***_**f**_ **(mm** **s**^−**1**^**)**	**Test duration (h)**	**Final bed state (−)**	***t***_**i**_**(h)**	***T***_**H**_**(h)**	***T***_**L**_**(h)**	Fraction of initial EPS in ripple after test* **(%)**
1	0 (D)	250	432	422	4	R	—	—	—	—
2	1 (D)	145	655	650	4	F	—	—	—	—
3	0.5 (W)	240	451	433	4	F	—	—	—	—
4	0.25 (W)	250	435	429	4	F	—	—	—	—
5	0.125 (W)	240	411	434	9	R	—	—	—	—
6	0.125 (D)	247	397	378	9	R	7.9	—	—	25
7	0.063 (D)	247	397	365	9	R	4.3	—	—	9
8	0.063 (D)	254	427	439	66	R	2.7	115.2	92.2	0
9	0.031 (D)	251	414	390	73	R	1	35.5	33	0
10	0.016 (D)	255	440	434	7	R	0.4	6.9	5	19
11	0 (D)	252	424	403	24	R	0.1	1.1	1.3	—

EPS, extracellular polymeric substances; F, flat bed; R, rippled bed.

Tests that have EPS mixes specified as ‘(D)’ were mixed dry for that particular run (percentage corresponds to dry mix) and those specified as ‘(W)’ were mixed wet and based on the successive dilutions of the 1% mix (see methods/preparation of bed). *U*_i_ and *U*_f_ are the initial and final depth-averaged current speeds determined from Ultrasonic Doppler Velocimetry Probe measurements and *t*_i_, *T*_H_ and *T*_L_ are the values obtained by fitting to equation (1) when abiotic equilibrium dimensions are assumed (*H*_e_=12 mm, *L*_e_=106 mm).

^*^Determined by carbohydrate analysis based on glucose equivalents.
