# Supplementary material for: The pervasive role of biological cohesion in bedform development
Source: Nat Commun. 2015 Feb 6;6:6257. doi: 10.1038/ncomms7257 (PMC4347294; doi:10.1038/ncomms7257)
Supplement: Supplementary Information — Supplementary Figures 1-2, Supplementary Table 1 and Supplementary Notes 1-2. [file ncomms7257-s1.pdf]

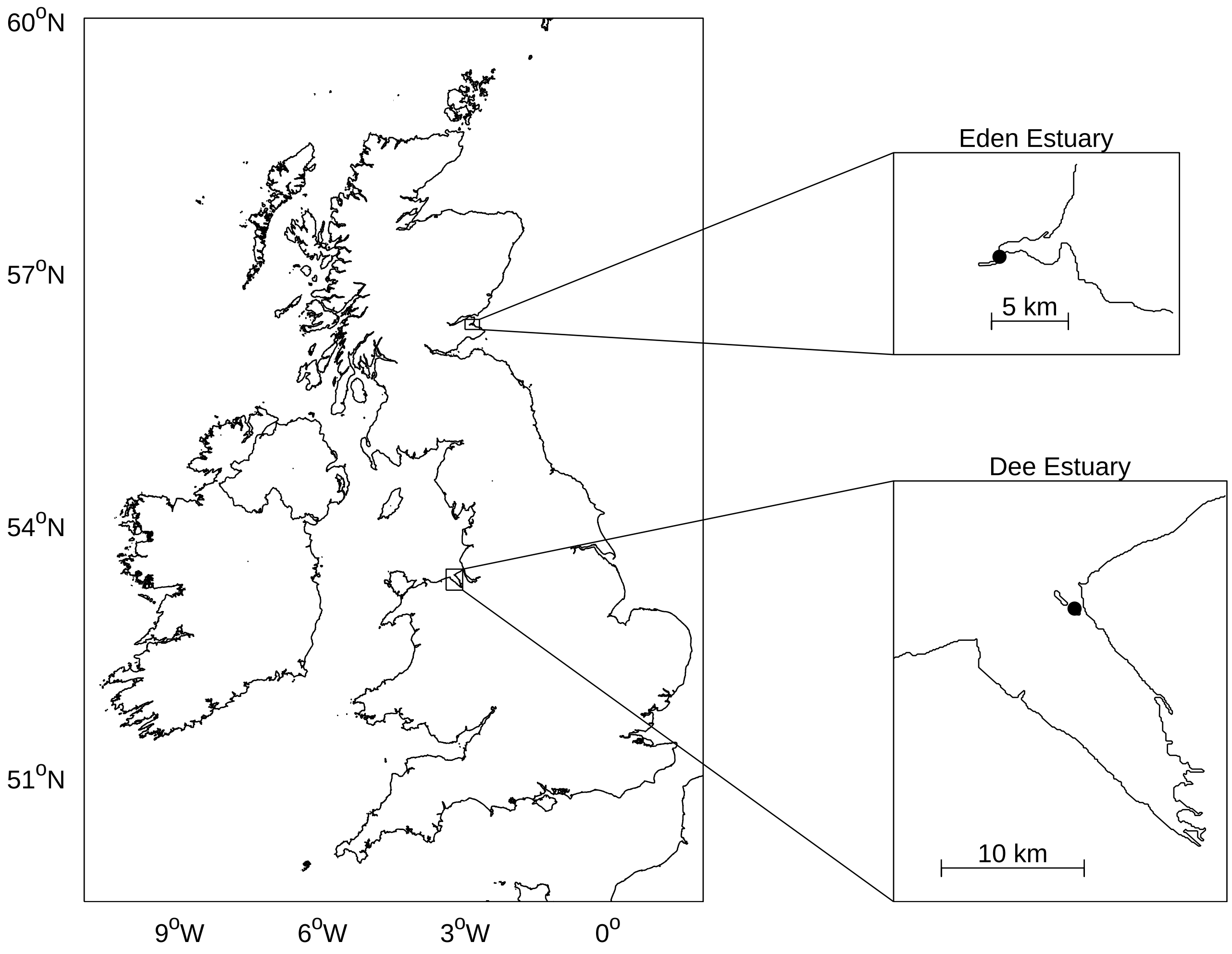

Supplementary Figure 1 | Map of the UK showing the location of the Dee Estuary and Eden Estuary sample sites.

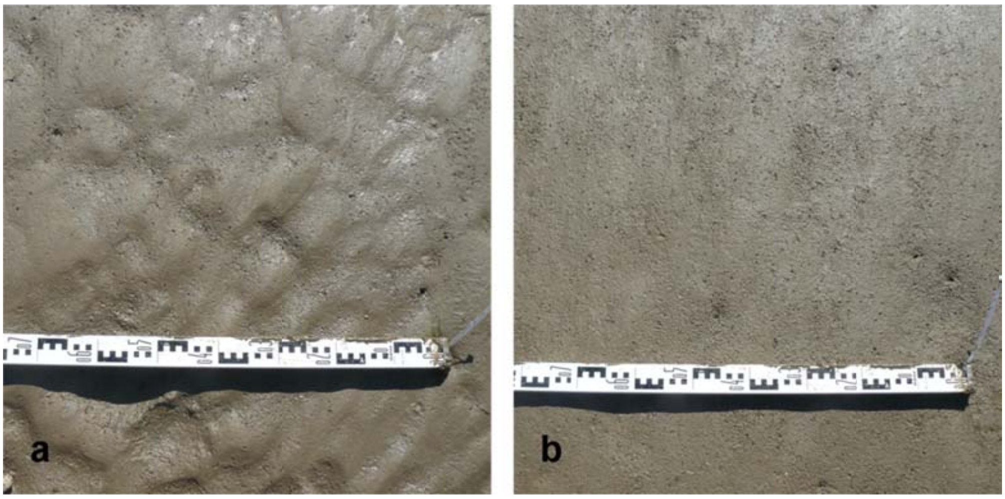

Supplementary Figure 2 | Beds in the vicinity of sample sites in the Dee Estuary. (**a**) site 1 and (**b**) site 2, the scale on the ruler is in cm.

Supplementary Table 1 | General estuary characteristics

	Dee	Eden
Area (km^2)	131	8
Mean tidal range (m)	7-8	4.3
Mean river discharge (m^3/s)	31	4

Supplementary Note 1: The Dee Estuary

The Dee Estuary is a large tidally-dominated estuary located on the Welsh-English border (Suppl. Fig. 1 and Suppl. Table 1). It has two deep channels, the Welsh Channel and the Hilbre Channel, which are subtidal, and three rocky outcrops near the mouth, Hibre Island, Little Hilbre Island, and Little Eye, which are islands at high water. The samples (site 1: 53°22’26.4”N 3°12’36”W; site 2: 53°22’26.9”N 3°12’35.9”W) were collected, 16 m apart near a gully, on the intertidal flats between Little Hilbre Island and Little Eye. This area had a depth of ~ 2 m at high water, and the median grain size of the bed sediment was 0.233 mm. The samples were taken at low tide on 31/05/2013. The beds in the vicinity of sites 1 and 2 are shown in Suppl. Figs. 2a,b. The bed was close to flat for site 1 and completely featureless for site 2.

Supplementary Note 2: The Eden Estuary

The Eden Estuary is a small tidally-dominated estuary located between St. Andrews and the Forth of Tay (Suppl. Fig. 1 and Suppl. Table 1). The sample site (56°21’29.4”N 02°53’20”W) was in the upper intertidal reaches of the estuary, about 10 m from the main channel. This area had a depth of ~ 2 m at high water, and the median grain size of the bed sediment was 0.277 mm. The sample was taken from a surface scrape (flask frozen in tact in-situ as 1 cm^2 block) at low tide on 11/10/2013. No picture is available of the bed at the site, but the bed was rippled.
